# Design Methodology for a Magnetic Levitation System Based on a New Multi-Objective Optimization Algorithm

**DOI:** 10.3390/s23020979

**Published:** 2023-01-14

**Authors:** Igor Reznichenko, Primož Podržaj

**Affiliations:** Faculty of Mechanical Engineering, University of Ljubljana, 1000 Ljubljana, Slovenia

**Keywords:** magnetic levitation, magnetic field sensors, multi-optimization algorithm, system design, controller tuning

## Abstract

Multi-objective (MO) optimization is a developing technique for increasing closed-loop performance and robustness. However, its applications to control engineering mostly concern first or second order approximation models. This article proposes a novel MO algorithm, suitable for the design and control of mechanical systems, which does not require any order reduction techniques. The controller parameters are determined directly from a special type of rapid analysis of simulated transient responses. The case study presented in this article consists of a magnetic levitation system. Certain difficulties such as the nonlinearity identification of the magnetic force and duo magnetic field sensor scheme were addressed. To point out the advantages of using the developed approach, the simulations as well as the experiments performed with the help of the created algorithm were compared to those made with common MO algorithms.

## 1. Introduction

Any engineering system goes through a design stage. A general rule is to try to resolve as many obstacles as possible during the design stage—this includes controller tuning. Certain circumstances may exist that interfere with online tuning. For example, the plant may have to be put offline. This may cause unnecessary stalls for a production line, which this particular system is a part of, which, depending on the process at hand, could be costly. Another case may be a long loop time—some processes in industry take a matter of days or longer. During the tuning process, it is naturally required to have several step-responses for a better quality of the tune. Finally, it is beneficial to have an idea of a good tune before the final implementation, which may lead to a better optimization overall.

The problem of applying and optimizing a proper controller is one of the central tasks of engineering. Whether this is a classic PID controller or a nonlinear one, the goal is to help the physical process at hand run within the needed boundaries and properties. A revisit of the classic control theory and a re-evaluation of its possibilities with modern computing powers provides for a new controller tuning method and a powerful tool for design optimization. Our method of controller tuning deals with a plant in the design stage. An algorithm was developed which performs a search in the parameter space with all the main transient response characteristics being rapidly estimated by it. It is based on fast computations of the inverse Laplace transform and curve analysis of the continuously generated stochastic Laplace images.

The idea behind this method appeared while working with a magnetic levitation system. A demand for a more efficient tune of a PID controller during the design stage led to the development of the algorithm described in this work. PID controllers are widely used in various industrial applications. The effectiveness of such controllers depends on tuning, which is essentially an optimization problem. To address this task, a number of tuning methods have been developed ever since the appearance of these controllers [1,2]. Since this system is open-loop unstable, experimental methods are difficult to implement, and it can be frustrating to tune it manually without a model. On the one hand, classic control has powerful tools for system design such as root locus, frequency response method, etc. [3,4]. However, the developed algorithm is based on a different approach. In a situation where the developed system’s model is known—fast computations of the inverse Laplace transform are used to provide lots of information about the studied system’s behavior. Whether it is the classical partial fraction expansion [5] or a numerical method [6,7,8] (as long as it is fast enough), combined with the developed algorithm, they make for a unified framework for designing and tuning closed-loop controlled mechanical systems.

The goal of this work was to explore this possibility and present an overview of such an approach. The developed algorithm is suitable for multi-objective optimization. Since a transient response of a mechanical system has a few characteristics that may be important for the designer, controller tuning can be formulated as a multi-objective problem. The developed algorithm is compared with a common multi-objective algorithm NSGA-II using Pareto-optimal front. At the same time, we discuss the challenges of the magnetic levitation system itself such as the identification of the magnetic nonlinearity. A detailed physical background is given in order to obtain the system model. At the end, we show how this approach is a foundation of a larger problem of system design, as this method can be applied to solve an inverse problem of finding the optimum plant parameters for the desired transient response characteristics.

## 2. Materials and Methods: The Magnetic Levitation System

The magnetic levitation set-up involves a well-known phenomenon often used for control system studies. Recently, it has become important in a very wide range of industrial applications where magnetic suspension techniques can be profitably applied. The best known ones are high-speed ground transportation [9,10] and high-speed bearings with reduced noise and friction [11,12].

**Figure 1:**
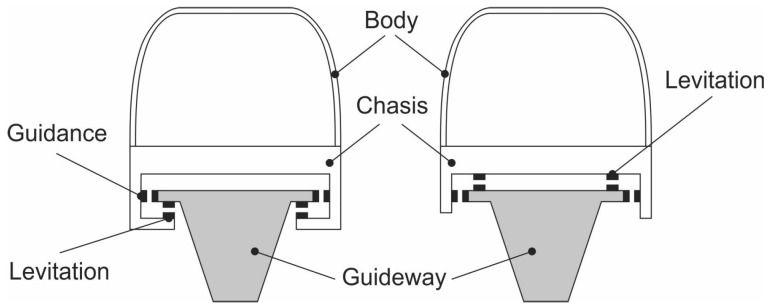
Two possible levitation setups (attraction-(**left**), repulsion-(**right**)) [9,14].

Levitation can in general be achieved in two ways. The first one is using AC in a primary coil. As a result a current is induced in a secondary coil, which is repelled from the primary one [13]. The height of the levitation can be controlled by amplitude and/or frequency of the AC. The second method is using DC current in the primary coil and a permanent magnet or a piece of ferromagnetic material as the levitated object. If a permanent magnet is being used, both attraction and repulsion are feasible. If however a ferromagnetic material is being used for levitation, only attraction is possible. From a control theory point of view it makes a difference whether attraction or repulsion is used in a magnetic levitation project (see Figure 1).

**Figure 2:**
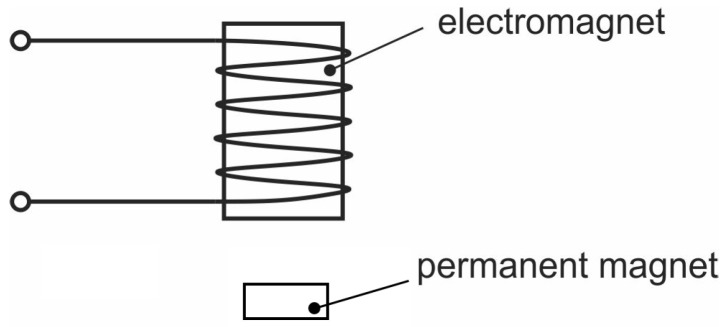
Scheme of a magnetic levitation system.

In the case of repulsion, the open loop system is stable. If, however, attraction is being used, the system is open loop unstable and is useless without a proper closed-loop design. In order to study magnetic levitation control, various experimental setups have been used  [15,16] but the most commonly used experimental setup is the one shown in Figure 2 [17,18].

The main goal of such systems is to make the permanent magnet levitate at a desired height. Various approaches known from control theory have been used for this purpose (root locus [19,20], state space [21], disturbance rejection control [22], fuzzy control [23], sliding mode control [24], fuzzy sliding mode control [25], robust control [26], neural network control [27], various nonlinear approaches [28,29]). The authors of [30] deal with dynamical uncertainties and exterior perturbations in a magnetic levitation system using a real-time prescribed performance control. This allows for chattering reduction and faster convergence to the equilibrium point. In [31], an analytical method using Lagrange equations for the analysis of magnetic levitation (MagLev) systems is proposed. This provides for an interesting MagLev model which distinguishes the primary and induced currents and also the equilibrium height of the levitating object on the input voltage through the mutual inductance of the system.

**Figure 3:**
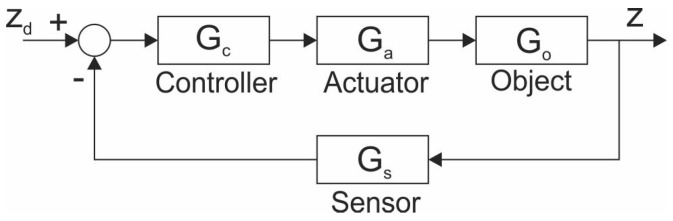
The basic block diagram of the system.

Before we discuss the designed controller tuning and system design method, let us describe the constructed magnetic levitation system. Let us start with the development of the system’s model. To do that, we use the classic block diagram representation shown in Figure 3.

The $z_{d}$ is the desired position of the permanent magnet (input signal), while *z* is the actual position (output signal). The $G_{c},\;G_{a},\;G_{o}$ and $G_{s}$ are the transfer functions of the corresponding parts of the experimental setup. Application of block diagrams leads to very descriptive relationships, especially in the case of automatic feedback systems. To acquire the model of the system we need to determine all of the expressions behind these blocks.

### 2.1. Object

By far the most difficult element to model is the object. Its input signal is the voltage $U_{c}$ on the coil and the output signal is the position of the magnet *z*. First, we should analyze the force acting upon a permanent magnet in a magnetic field.

#### 2.1.1. Determination of the Magnetic Force

**Figure 4:**
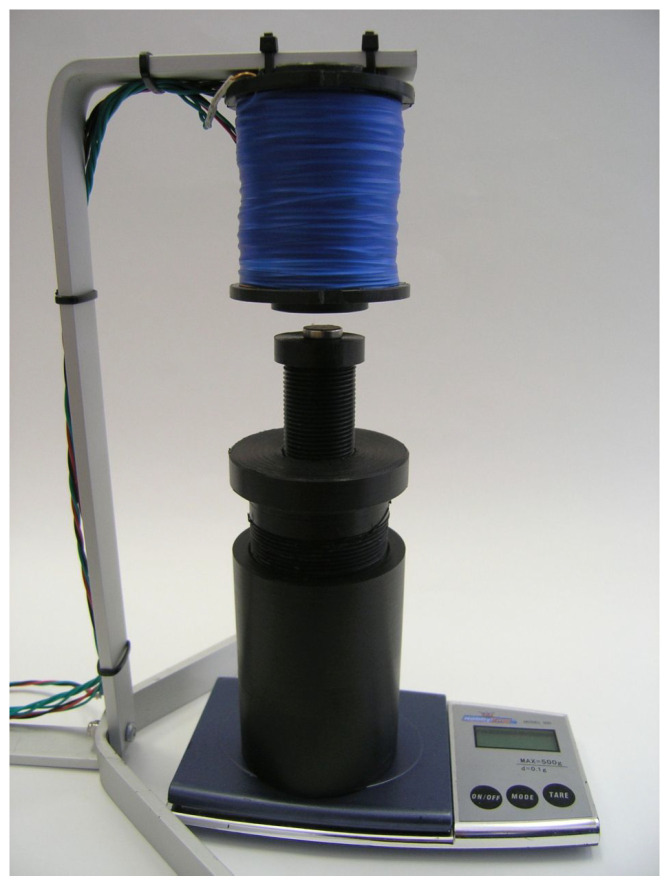
Experimental determination of the magnetic force.

To determine the magnetic force $F_{m}$, we used the experimental setup shown in Figure 4. An electromagnetic coil is a length of wire wound in a joined sequence of concentric rings through which an electric current *i* flows. The magnetic field B (or its component $B_{z}$) of a single ring can be obtained by the application of the Biot–Savart law,

(1) $$B_{z}=\frac{\mu_{0}}{2\pi }i\frac{\pi R^{2}}{{\left(z^{2}+R^{2}\right)}^{3/2}},\;{\frac{\partial B_{z}}{\partial z}}_{coil}=-\frac{3}{2}\cdot \frac{\mu_{0}iR^{2}z}{{\left(z^{2}+R^{2}\right)}^{5/2}},$$

where $\mu_{0}=4\pi \cdot 10^{-7}$ H/m is the magnetic permeability of vacuum and *R* is the radius of the coil’s winding.

A permanent magnet can be modeled as a collection of many microscopic current loops (magnetic dipoles). The net effect of these small current loops is a surface current $i_{m},$ which is called the Amperian current [32]. Let the current loop (magnetic dipole) have a magnetic moment of $\mu =\left(\mu_{x},\mu_{y},\mu_{z}\right)$ and be in a uniform magnetic field $B=\left(B_{x},B_{y},B_{z}\right).$ If the loop is small enough, then the torque acting on such a loop is given by a simple expression [33]:

(2) τ=μ×B;

therefore, the force F acting on a magnetic dipole in this field is the gradient of the potential energy associated with this torque:

(3) $$F=-\nabla U_{B}=-\nabla \left(-\mu \cdot B\right)=\nabla \left(\mu \cdot B\right).$$

As magnetic dipole moment μ has only a vertical component ($\mu_{z}$ in this case), which is constant, we can write the vertical component of the force $F_{z}$ as follows:

(4) $$F_{z}=\mu_{z}\frac{\partial B_{z}}{\partial z}.$$

The magnet was attached to the plastic pedestal using adhesive tape. The pedestal was made with a screw so that it is easy to put the magnet at a desired height *z*. The pedestal was put on scales. When there is an attractive force between the electromagnet and the permanent magnet, the reading on the scales is lower. In this way, a matrix of possible heights *z* (measured from the lower end of the electromagnet) and electrical currents *i* through the coil was formed. The results are given in Table 1.

The relationship between these variables can be obtained using any available curve fitting tool. The data from Table 1 must be imported and then fitted using a custom function *f* in the form of $F_{m}=f\left(i,z\right)$, where *i* and *z* are independent variables of current and coordinate and $F_{m}$ is the dependent variable of magnetic force. In many papers dealing with the magnetic levitation, the force of the coil is approximated by the formula $F_{m}=const\cdot i/z^{2}$. This yields acceptable results in some cases but in this research we paid extra attention to the accuracy of the acquired expression.

For a real magnetic coil, it is often quite difficult to acquire an explicit expression of the magnetic field. One could be surprised by the scale of difficulties appearing on this path. For objects with an infinite dimension, explicit formulas usually do exist since it is possible to perform a limit passage. Another possibility is the geometry of the given current contour having a symmetry axis (like the solenoid when the field is calculated at some point on the coaxial line of the magnet). One of the obstacles to consider is the fact that the majority of the formulas are for a magnetic force applied to a material point. In the case of a magnetic force applied to a permanent magnet things are even more complicated due to the shapes of the interacting magnetic objects as effects such as mutual inductance have to be taken into account. In the case of non-simplest volumetric bodies it is often impossible to integrate the expression (1) analytically. Numerous approaches exist to simplify this process such as Maxwell’s Method [34]. This is why, in engineering, numerical approaches such as the finite elements method or the boundary integral equations method are often implemented. However, in our research we require an explicit expression for the force applied to the permanent magnet.

It is safe to assume that a model with an explicit formula representing a closer geometry to the original set up would provide a better fit for the given measured data; we show that in Table 2. Here, we establish that one of the best fits for our data is, in fact, the expression using (4):

(5) $$F_{z}=-a\cdot \frac{3\mu_{z}}{2}\cdot \frac{\mu_{0}iR^{2}z}{{\left(z^{2}+R^{2}\right)}^{5/2}},$$

where $\mu_{0}=4\pi \cdot 10^{-7}$ H / m is the magnetic permeability of vacuum, $\mu_{z}$ is a magnetic dipole moment of the permanent magnet and *a* is a constant related to the length of the coil *L* and the winding turns per unit length *n*. The length of the coil is L=54mm, while the mean radius of the coil’s winding is R=37mm. The permanent magnet has a form of a cylinder with 4 mm radius and 5 mm height. For a neodymium magnet of this size, the vertical component of the magnetic moment can be estimated to have a value of $|\mu_{z}|=0.49\;A\cdot m^{2}$. As a means of comparison we chose the commonly used functions:

(6) $$F_{z}=a\frac{i}{z},\;\;F_{z}=a\frac{i}{z^{2}},\;\;F_{z}=a\frac{i}{z^{3}}.$$

Using curve fitting software, we determined the value of parameter *a* and also the plausibility of the fit as a sum of squared errors (SSE). The results are summarized in Table 2.

The fit using expression (5) has a sum of squared errors of $5.6\times 10^{-5}$, which is by two orders of magnitude lower than the most used fit $ax/y^{2}$. Therefore, expression (5) provides for a much better approximation.

#### 2.1.2. Experimental Determination of Coil Resistance and Inductance

**Figure 5:**
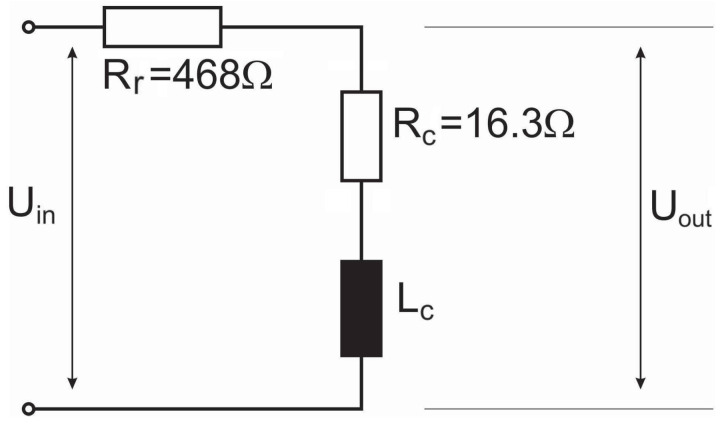
Input voltage $U_{in}$ and output voltage $U_{out}$ in a scheme to determine the coil’s inductance $L_{c}$.

The electromagnet used in our experiment was a reel of PU enamelled, unjacketed copper wire. Its length was 230m and the cross sectional area was $0.246\;{\mathrm{mm}}^{2}$. The resistance of the coil $R_{c}$ can easily be measured using a multimeter. In the case of our coil it was $R_{c}=16.3\;\Omega$. In order to determine the inductance of the coil $L_{c}$, a resistor $R_{r}=468\;\Omega$ was added in series to the coil as shown schematically in Figure 5.

The ratio between output voltage $U_{out}\left(t\right)$ and input voltage $U_{in}\left(t\right)$ can be obtained by a simple voltage divider rule,

(7) $$\frac{U_{out}\left(j\omega \right)}{U_{in}\left(j\omega \right)}=\frac{Z_{c}}{Z_{r}+Z_{c}}=\frac{R_{c}+j\omega L_{c}}{R_{r}+R_{c}+j\omega L_{c}}.$$

The ratio of amplitudes of the output and input voltages is, therefore, given by:

(8) $$\frac{U_{out}}{U_{in}}=\frac{|U_{out}\left(j\omega \right)|}{|U_{in}\left(j\omega \right)|}=\frac{\sqrt{R_{c}^{2}+{\left(\omega L_{c}\right)}^{2}}}{\sqrt{{\left(R_{r}+R_{c}\right)}^{2}+{\left(\omega L_{c}\right)}^{2}}}.$$

By a simple algebraic manipulation, we get the following result:

(9) $$L_{c}=\frac{1}{\omega }\sqrt{\frac{U_{out}^{2}{\left(R_{c}+R_{r}\right)}^{2}-U_{in}^{2}R_{c}^{2}}{U_{in}^{2}-U_{out}^{2}}}.$$

**Figure 6:**
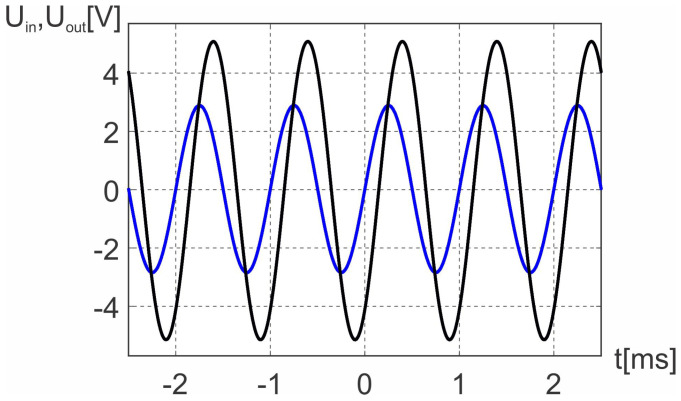
Input voltage $U_{in}$ (black) and output voltage $U_{out}$ (blue) for the circuit shown in Figure 5.

The actual waveforms for both $U_{in}\left(t\right)$ and $U_{out}\left(t\right)$ are shown in Figure 6.

For the signal $U_{in}=5.12\;V$ and ω=6280rad/s we get the $U_{out}=2.87\;V$. Finally, using Equation (9) we get $L_{c}=52.1\;\mathrm{mH}$.

#### 2.1.3. The Transfer Function of the Object

With all the parameter values known, we can now create the model of the object, the input of which is the voltage while the output is the current i(t).

(10) $$G_{coil}\left(s\right)=\frac{\hat{I}\left(s\right)}{\hat{U_{c}}\left(s\right)}=\frac{1}{Ls+R_{c}}.$$

If we neglect the viscous resistive forces of air, the only forces acting on the magnet are the gravity and the magnetic force. The movement in the vertical direction (*z*-axis) can then be described by the following differential equation:

(11) $$m\frac{d^{2}z}{dt^{2}}=F_{z}-mg,$$

where m=3grams is the mass of the magnet, *g* is the gravitational acceleration and the force $F_{z}$ is given by the expression (5). Let $\hat{z}\left(t\right)$ be the relative change of coordinate *z* from the initial state $z_{0}$:

(12) $$z\left(t\right)=z_{0}+\hat{z}\left(t\right).$$

Zero initial value $\hat{z}\left(+0\right)=0$: satisfies the rule for differentiation of originals for the Laplace Transform [5]. It is necessary to linearize the force $F_{m}$ in Equation (11). We select the operating point to be the equilibrium state at $z_{0}=-25\;\mathrm{mm}$. Using (5) to calculate the initial current from equation $F_{z}=mg$ we get $i_{0}=0.23\;A$. The initial voltage is $U_{c0}=i_{0}R_{c}=3.67\;V$.

We expand the function $F_{z}$ in a Taylor series at the point $\left(i_{0},z_{0}\right)$, where $\hat{i}=0,\hat{z}=0$. Since the operating point is chosen in a way that $mg=F_{z}\left(i_{0},z_{0}\right)$ we rewrite the Equation (11) as

(13) $$m\frac{d^{2}\hat{z}}{dt^{2}}=\frac{\partial F_{z}}{\partial z}\left(i_{0},z_{0}\right)\cdot \hat{z}+\frac{\partial F_{z}}{\partial i}\left(i_{0},z_{0}\right)\cdot \hat{i},$$

where

(14) $$\frac{\partial F_{z}}{\partial z}\left(i_{0},z_{0}\right)=-\frac{3\mu_{z}R^{2}i_{0}}{2}\frac{R^{2}-4z_{0}^{2}}{{\left(z_{0}^{2}+R^{2}\right)}^{7/2}}$$

and

(15) $$\frac{\partial F_{z}}{\partial i}\left(i_{0},z_{0}\right)=-\frac{3\mu_{z}R^{2}}{2}\frac{z_{0}}{{\left(z_{0}^{2}+R^{2}\right)}^{5/2}}.$$

The Laplace Transform of the Equation (13) is

$$s^{2}\hat{Z}\left(s\right)=a\hat{Z}\left(s\right)+b\hat{I}\left(s\right),$$

where

$$a=\frac{1}{m}\frac{\partial F_{z}}{\partial z}\left(i_{0},z_{0}\right)=-783.3,\;b=\frac{1}{m}\frac{\partial F_{z}}{\partial i}\left(i_{0},z_{0}\right)=39.9.$$

Note that, with this system and the direction of the *Z*-axis, *a* and *b* must be greater than 0. The transfer function of Equation (13) is, therefore:

$$G_{magnet}\left(s\right)=\frac{\hat{Z}\left(s\right)}{\hat{I}\left(s\right)}=\frac{b}{s^{2}-a}.$$

Combined with the expression (10) the resulting transfer function of our magnetic levitation system is

(16) $$G_{o}\left(s\right)=\frac{b/L_{c}}{s^{3}+\left(R_{c}/L_{c}\right)s^{2}-as-aR_{c}/L_{c}}=\frac{766}{s^{3}+313s^{2}-783s-2.45\times 10^{5}}.$$

### 2.2. Actuator

**Figure 7:**
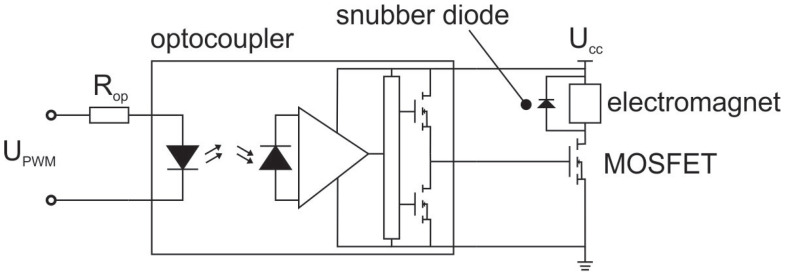
The scheme of the actuator.

As shown in Figure 3, an actuator is an element between the controller and the object. In our case, its primary role was to supply proper voltage and current for the electromagnet. It is shown schematically in Figure 7.

It was made of an optocoupler (LED—phototransistor pair and a MOSFET driver) and a MOSFET. The MOSFET was in series connection with the electromagnet. A snubber diode was added in order to suppress the transients, which appeared due to the pulse width modulated input signal $U_{PWM}$ (which is the output voltage of the controller shown in Figure 7). The maximum value of $U_{PWM}$ is equal to 3.33V. The voltage $U_{cc}$ was selected to be 16.2V, so that the maximum current through the electromagnet is 1A. The transfer function of the actuator is, therefore, equal to:

(17) $$G_{a}=\frac{16.2\;V}{3.33\;V}=4.86.$$

### 2.3. Sensor

**Figure 8:**
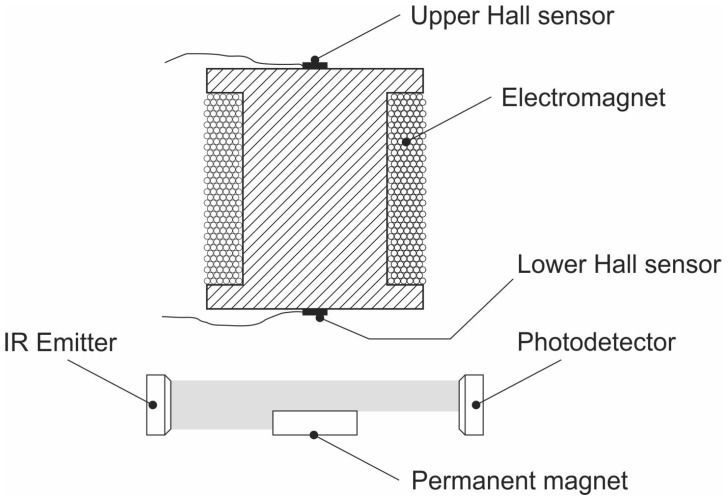
Possible sensors for detecting position of the permanent magnet.

The role of the sensor is to detect the actual position of the permanent magnet. In general it can be detected in two ways as shown in Figure 8.

**Figure 9:**
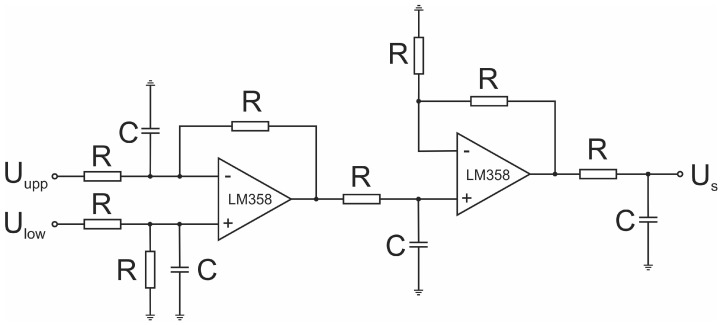
Schematic representation of a coil.

The first and the most common one is the application of a photo-emitter and a photo-receiver [17,25,28,35,36,37] or some similar arrangement based on optical means [22,38] The second one is the application of one or two Hall sensors [39,40]. Sometimes an inductive sensor is used [41,42]. In our case, a pair of SS49E Hall sensors have been used. The idea behind using a Hall sensor is to detect the magnetic field of the permanent magnet. When it moves closer to the electromagnet, an increase in the magnetic field can be detected. The problem is, however, that the electromagnet generates a magnetic field of its own as well. When the current through the electromagnet is changed due to the varying control signal, the sensor cannot tell if the magnetic field changed due to the changed current through the electromagnet or due to the movement of the permanent magnet. If a pair of such sensors is used (one at the upper and one at the lower end of the electromagnet), their output signals can be subtracted and the resulting signal is due to the magnetic field of the permanent magnet alone. In order to get this signal the circuit shown in Figure 9 is used.

It is composed of two operational amplifiers. The first one is used for subtraction and the second one for amplification. The circuit output for various distances of the permanent magnet is given in Table 3.

The dependence between the voltage $U_{s}$ and the permanent magnet position can then be obtained in a similar way as in the case of the magnetic force $F_{m}$. Using a curve fitting software, we get a reasonably good approximation with:

(18) $$U_{s}=f_{s}\left(z\right)=\frac{-1.707\times 10^{5}}{z^{4}}.$$

At this stage, we could also make linearization and get the $G_{s}$ denoted in Figure 3, but it is easier to include $z=f^{-1}\left(U_{s}\right)$ in the controller.

### 2.4. Controller

**Figure 10:**
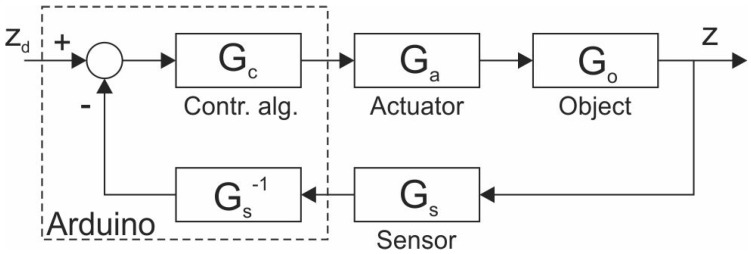
The role of the microcontroller (Arduino) in the system.

The role of the controller is played by the Arduiuno Due microcontroller. In reality, the block diagram of the whole system shown in Figure 3 should be modified as shown in Figure 10.

The microcontroller has two inputs. One is the sensor voltage $U_{s}$, which can be used to obtain the permanent magnet position *z* using

(19) $$z=f_{s}^{-1}\left(U_{s}\right)=-\sqrt[{4}]{\frac{1.707\cdot 10^{5}}{U_{s}}}.$$

The other one is the desired position of the permanent magnet $z_{d}$, which can be entered into a microcontroller via a Serial Monitor (serial communication with a PC). With the subtraction of *z* from $z_{d}$ we get the error signal. The error signal is the input for $G_{c}$ (block Contr. Alg. in Figure 3 and Figure 10). The control algorithm is the common PID algorithm. The control signal (output of the controller) is a pulse width modulated signal.

## 3. Materials and Methods: The Developed Algorithm

### 3.1. Problem Statement

Providing the underlying calculation method is fast enough, one can look at the tuning problem from a different angle. While the classical methods do exist and provide the user with satisfying results, sometimes they seem a little bit locked on to their sequence of actions. It is always a good idea to let the simulation “run free” in terms of possible perturbations, irregularities and parameter values. Real systems tend to yield slightly different results to simulations. This raises an important question—what is the “optimal” controller tuning? Should we stop when have reached acceptable transient response characteristic or is it beneficial to keep going to explore the system’s behavior over an area of controller parameters? By area we mean certain intervals within the parameter space (rectangles on a plane, certain cubic areas in 3-dimensions parameter space).

In the literature, the transient response analysis mostly comes down to analyzing the known characteristics of a first- or second-order-like mechanical system [43,44,45,46,47]. Usually, systems of higher order are approximated to it by known techniques. The convenience of the second-order-like system approximation, the simplicity of the action–result methodology of adjusting loop gains led to other techniques of step-response analysis being overlooked. Higher order systems do not have such an intuitive correlation between transient response parameters and controller gains. Therefore, a second-order approximation is commonly used.

In this section, we describe a new optimization algorithm and test it in a case of the magnetic levitation of a small cylinder with a PID controller as means of keeping it at a desired height. PID controllers are still one of the most commonly used controllers in industrial applications [1]. The idea behind them is intuitive—with the help of examples, one can understand the core principles without referring to the Laplace Transform. There are numerous ways of tuning a PID controller. Many of these methods involve the step-response method either by using a process model or an experiment. The output is measured or calculated as a function of time. By analyzing it, a new set of controller parameters is chosen. Many strategies exist in properly applying a PID controller. Ref. [48] suggests a heuristic algorithm using wavelets for online tuning of a gain adapted PID-controlled linear actuator. Permanent magnets are implemented as excitation with the aim of soft landing which increases reliable functionality and component life. In [49], another technique is utilized to achieve tracking and soft landing for electromagnetic actuators. Pre-action is employed to enable the system to avoid power saturation. For constrained processes, it was demonstrated that a PID with anti-windup is able to provide similar or even better results than model predictive control when certain solutions are considered [43]. Conditions on nonlinearity and uncertainty are addressed in [50] so that a high order affine-nonlinear system under an extended PID controller can be semi-globally stabilized with a fast rate of regulation error convergence.

First, let us unite the actuator and object into a single plant block. The resulting transfer function of the plant would be $G_{ao}\left(s\right)=G_{a}\left(s\right)\cdot G_{o}\left(s\right),$ and

(20) $$G_{ao}\left(s\right)=\frac{3723}{s^{3}+313s^{2}-783s-2.45\times 10^{5}}.$$

Due to negative coefficients of the polynomial in the denominator the system is unstable, hence a controller is needed. As seen in Figure 10, since $G_{s}\left(s\right)\cdot G_{s}^{-1}\left(s\right)=1$, we have a unity feedback control system. The transfer function of the PID controller in a parallel form is $G_{c}\left(s\right)=K_{p}+K_{i}/s+K_{d}\cdot s,$ where $K_{p},K_{i}$ and $K_{d}$ are positive real values. The resulting transfer function of the controlled magnetic levitation system T(s) ends up being

(21) $$\frac{A(K_{d}s^{2}+K_{p}s+K_{i})}{a_{3}s^{4}+a_{2}s^{3}+\left(a_{1}+AK_{d}\right)s^{2}+\left(a_{0}+AK_{p}\right)s+AK_{i}},$$

where $A=3723,\;a_{3}=1,\;a_{2}=312.9,\;a_{1}=-783.3,\;a_{0}=-2.45\cdot 10^{5}$. This transfer function is going to be the subject for testing our algorithm.

In order to obtain a step response of our system, first we multiply the transfer function (21) by 1/s and then perform the inverse transformation. Since the function (21) is in the form of a polynomial divided by a polynomial of degree lesser than the one in the nominator, then the inverse Laplace transformation of such a function is given by a partial fraction expansion [5]. Therefore, the explicit expression of the signal f(t) and its derivative $f^{\prime }\left(t\right)$ at any given point in time is presented.

We are going to show how, for this magnetic levitation system represented as a transfer function, our algorithm will provide numerous stable solutions while recording all the necessary step-response characteristics. An array with such data is created in the process which is later used for analysis and optimization. While we do provide the reader with a mathematical background, it is not required from a user to go in-depth into the inner workings of the inverse Laplace transform.

### 3.2. Description of the Algorithm

Mathematical optimization is a process of finding the best selection from a set of available options such as minimizing a function by choosing different input values. Usually, the input values are bounded. The type of domains and criteria for the best choice can vary largely depending on the type of problem. Therefore, these tasks are a significant part of applied mathematics. The algorithm starts with a calculation of random controller parameters within the limits.

- Let *M* be the number of simulations we would like to perform with a given transfer function.
- Assume $\left(K_{p,min},K_{p,max}\right)$, $\left(K_{p,min},K_{p,\max}\right)$ and $\left(K_{p,min},K_{p,max}\right)$ to be the limits of the controller parameters, then
  $$K_{p}=K_{p,min}+\left(K_{p,max}-K_{p,min}\right)\cdot \rho_{1},$$
  (22) $$K_{i}=K_{i,min}+\left(K_{i,max}-K_{i,min}\right)\cdot \rho_{2},$$
  $$K_{d}=K_{d,min}+\left(K_{d,max}-K_{d,min}\right)\cdot \rho_{3},$$
  where $\rho_{1},\;\rho_{2}$ and $\rho_{3}$ are uniformly distributed random numbers. This algorithm can scan any region of parameter space of interest, however for the initial search it is safe to assume that $K_{p,min}=K_{i,min}=K_{d,min}=0.$ The upper limit, however, requires more attention since the equipment at hand may not handle too large values of the controller parameters. For example, in our case the upper limit of the electrical current in the coil was around 1A, or, in terms of voltage—16.3V. It is easy to calculate the upper limits with the inverse Laplace transform of the expression
  (23) $$\frac{1}{\Delta z\;s}\left(K_{p}+\frac{K_{i}}{s}+K_{d}s\right)\frac{1}{Ls+R},$$
  where Δz is the height (difference) a magnet needs to move up by. By substituting the t=0 in the resulting expression of f(t), one may find whether the modeled response would correspond to that of a real plant.

**Figure 11:**
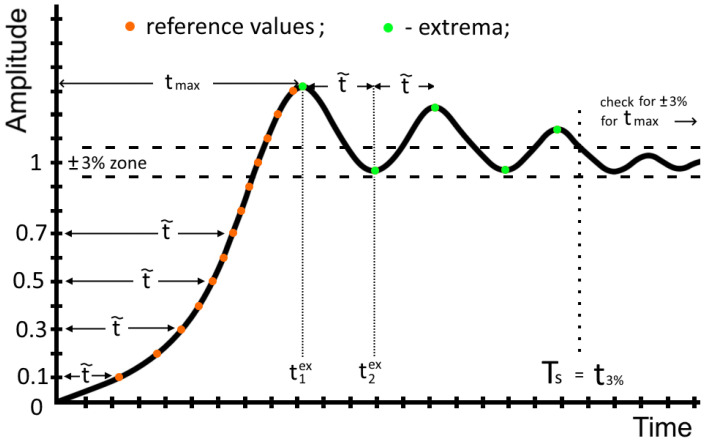
The algorithm analyses a simulated transient response. The orange points represent the times when the output signal crosses a new reference value. The green points are the signal’s extrema. The key parameter of this process is the current recorded reference time $\tilde{t}$. The two mechanisms for when this value changes are described (see the algorithm’s description).

For the given set of controller parameters, the algorithm continues by finding the roots $\alpha_{m}$ of the polynomial in the denominator P(s), which in our case is $s(a_{3}s^{4}+a_{2}s^{3}+\left(a_{1}+AK_{d}\right)s^{2}+\left(a_{0}+AK_{p}\right)s+AK_{i}).$ If any of the roots are in the right-half plane, then the solution is unstable and we move to a new simulation. However, if all the roots are in the right-half plane the algorithm proceeds with the analysis of the resulting step-response signal. Before we start, let us introduce the variables and their initial values (see Figure 11).

**Figure 12:**
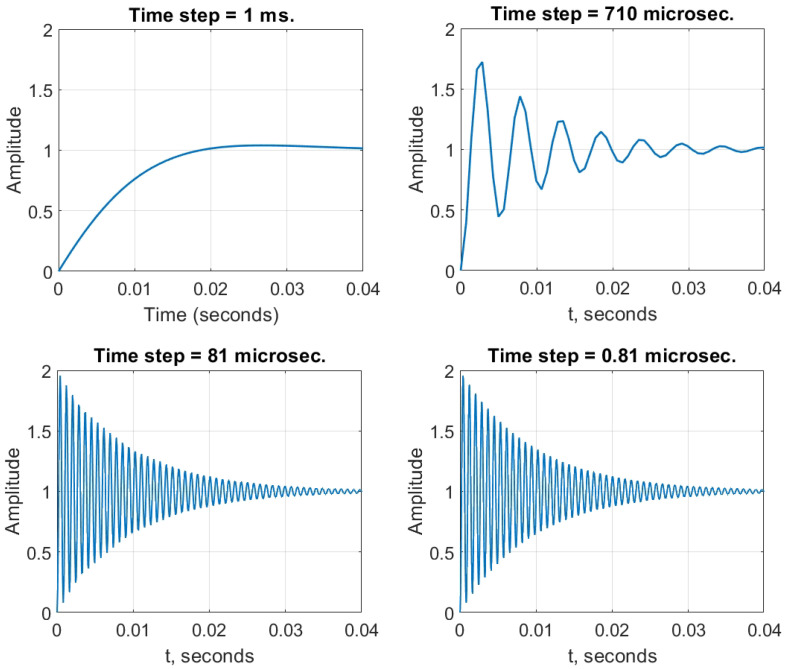
A showcase of modelling a mechanical system without satisfying the Nyquist theorem. For some mechanical systems it may be of great importance to distinguish oscillatory step responses. Such oscillations may, for example, result in unnecessary sideways oscillations which may result in complete loss of control over the object. The developed algorithm, however, requires no prior knowledge about the time scale of the process involved as it determines the appropriate time step Δt=2 microseconds automatically.

- *t*– current time that the algorithm uses for calculations. Starts from zero;
- f(t)—the value of the step response signal at the current time *t*;
- $f^{\prime }\left(t\right)$—the value of the step response signal’s derivative at the current time *t*;
- Δt—the time step. This value changes as the algorithm progresses, depending on the step response. You can safely start with a very low value as the algorithm quickly finds the suitable time step for your process. For example, if one can ignore the changes occurring on the time scale of 1 ns, then the initial value can be Δt=1 ns.
- $\tilde{t}$—key parameter, the current recorded reference time. The two mechanisms for when this value changes are described later;
- n=0—how many decimal amplitude values the signal has crossed. An auxiliary counter needed for the first mechanism for detecting $\tilde{t}$;
- k=0—an auxiliary counter needed for the second mechanism for detecting $\tilde{t}$. Number of local extremums;
- $t_{min}$—the minimum out of all recorded reference times $\tilde{t}$. Needed for the calculation of the time step Δt. This parameter helps the algorithm to distinguish the possible rapid oscillations (see Figure 12);
- N=100—a positive integer regulating how fine do we divide the shortest reference time $t_{min}$ to calculate the Δt. Increase this parameter for additional accuracy;
- $t_{max}$—the largest reference time $\tilde{t}$ used as a verification for determining the settling time $T_{s}$;
- $t_{k}^{ex}$—the time of a local extremum;
- $f_{k}^{peak}$—the amplitude of a local extremum;
- $t_{3\%}$—the time when the signal reached the ±3% range;
- $T_{s}=0$—the settling time of the current curve;
- PO—percentage overshoot;
- $OA_{k}$—the amplitude difference between neighboring oscillations;
- $OA_{max}=0$—largest amplitude between neighboring oscillations. This helps to understand the scale of oscillations of the signal. If the number of local extremums k≤1, then this value is not defined.

Now, using pseudo code, let us describe the inner workings of the algorithm (see Algorithm 1). The algorithm scans the controller parameters’ space while performing the fast inverse Laplace transform calculations. It provides us with an explicit formula for the response of the initial system to a unit step signal. At the same time, it determines important signal parameters which are the number of oscillations before settling time, peak value, overshoot peak time, etc. For example, these signal data allow us to sort out highly oscillating responses.

One important feature of the designed algorithm is the fact that it automatically takes into account the Nyquist–Kotelnikov–Shannon theorem. This theorem specifies that a sinusoidal function in time or distance can be regenerated with no loss of information as long as it is sampled at a frequency greater than or equal to twice the frequency of oscillation. As shown above, the second mechanism of determining $\tilde{t}$ ensures the appropriate level of time resolution. In is important to keep this in mind while using some of the commercially available software. Let us show how, using a software without directly controlling the sampling rate may result in a wrong representation of a step response of a given system. To demonstrate the announced effect, we crafted a special transfer function of the same type as before (21), but $A=4.028\times 4.86\times 10^{5},a_{3}=1,a_{2}=207.7,a_{1}=-1257,a_{0}=-2.61\times 10^{5};$ and $K_{p}=14,K_{i}=1.6,K_{d}=30.$ Assume one needs to know the step response characteristics of the current process represented as this transfer function.

For this demonstration, the time step Δt was manually controlled (see Figure 12). If we select the time step to be larger, like on the first plot—the transient response is a smooth curve with little to no overshoot. However, once we start decreasing the time step, oscillations emerge. At first these oscillations are angular. This indicates that the time moments at which we calculate the signal response miss some of the oscillations. This becomes obvious when, after the time step of 81 microseconds (the last two plots), new oscillations stop emerging and no new peaks appear. This means that we have reached the needed accuracy to truly represent this system’s step response. On its own, the algorithm chose the appropriate time step in less than 81 microseconds (for this process the last recorded time step was Δt=2 microseconds). This feature makes the developed algorithm adaptive to different time scales.
**Algorithm 1** Processing of the given signal. Using the following algorithm, we gather large statistical dataPart 1.**while**$T_{s}=0$▹ continue until the settling time $T_{s}$ is determinedt←t+Δt.▹ increasing time by ΔtThe first mechanism of recording $\tilde{t}$ (orange dots on Figure 11):**if**$n\leq \left[f(t)/0.1\right]<n+1$**then**▹ where [ ] represents the floor function    $n++;\;\tilde{t}=\left(2t+\Delta t\right)/2;$ ▹ when the signal crosses decimal values a reference time is recorded (orange dots on Figure 11).    **if** n+k=1 **then** ▹ if this is the first a reference time $\tilde{t}$ is recorded, then        $t_{min}=\tilde{t};\;t_{max}=\tilde{t};$ ▹ assign this value to both $t_{min}$ and $t_{max}$    **else**        $t_{max}=\tilde{t};$ ▹ renewing only the maximum reference time    **end if****end if**$\Delta t=t_{min}/N;$▹ calculate time stepSee Part 2 for further explanation.Part 2.The second mechanism of recording $\tilde{t}$ involves an extremum search (green dots on Figure 11). Since the function f(t) is continuous, a moment of time when the derivative $f^{\prime }\left(t\right)$ changes sign implies a local extremum.**if**$f^{\prime }\left(t\right)\cdot f^{\prime }\left(t-\Delta t\right)<0$**then**▹$f^{\prime }\left(t\right)$ changes sign, so a local extremum occurs in (t−Δt,t)    $t_{k}^{ex}=\left(2t+\Delta t\right)/2;\;k++;$ ▹ record the local extremum time and the number of them    $f_{k}^{peak}=f\left(t_{k}^{ex}\right);$ ▹ amplitude at the extremum    **if** $f_{k}^{peak}\geq f_{k-1}^{peak}$ **then** ▹ we have found a larger peak        $PO=(f_{k}^{peak}-1)\cdot 100\%$ ▹ percentage overshoot    **end if**    **if** k>1 **then** ▹ if there are multiple extremums        $OA_{k}=\left|f_{k}^{peak}-f_{k-1}^{peak}\right|$ ▹ the amplitude difference between the neighbor oscillations        $OA_{max}=\max\left(OA_{k},OA_{max}\right)$ ▹ recording the maximum OA    **end if**    **if** k=1 **then** ▹ if this is the first extremum. This is done so that the $t_{max}$ does not increase uncontrollably.        $t_{max}=\max\left(t_{max},t_{k}^{ex}\right);$ ▹ the condition k=1 is needed so that $t_{max}$ does not increase uncontrollably    **else**        $\tilde{t}=t_{k}^{ex}-t_{k-1}^{ex};$▹ record the time between oscillations for additional accuracy (Nyquist theorem)        $t_{min}=\min\left(t_{min},\tilde{t}\right);\;t_{max}=\max\left(t_{max},\tilde{t}\right);$ ▹ in case this time is lower that the current $t_{min}$ or larger than the current value of $t_{max}$    **end if****end if**$\Delta t=t_{min}/N;$▹ calculate time step*Cont.*At last, let us describe the test for finding $T_{s}.$**if**f(t)∈[0.97,1.03]**then**▹ the signal appeared in the 3% range    $t_{3\%}=t;\;m=0;$ ▹ record the time and assign zero to the logic parameter *m* needed for the following cycle    **while** $t\leq t_{3\%}+t_{max}$ AND m=0 **do** ▹ starting from $t_{3\%}$ for the time length of $t_{max}$ we check for the 3% criteria        t=t+Δt        **if** f(t)∉[0.97,1.03] **then** ▹ the signal exited the 3% area           m=−1; ▹ therefore, the test for settling time stops and the algorithm resumes its work        **end if**    **end while**    **if** m≠−1 **then** ▹ if the 3% condition did not break once during this test        $T_{s}=t_{3\%}$ ▹ we have found the settling time    **end if****end if**

## 4. Results and Discussion

### 4.1. Multi-Objective Optimization

The developed algorithm returns the values of settling time $T_{s}$, percentage overshoot PO, number of local extremums *k* and the largest amplitude between the oscillations $OA_{max}$ in the form of Table 4. Using this table, one can easily arrange the column with a certain step-response characteristic (overshoot, for example) in ascending order to chose those controller parameters that yielded the preferred value (in our case, the lowest overshoot). The user is provided with enough information to sort out oscillating responses or understand their magnitude, using the parameter $OA_{max}$. The reason we try to avoid too much overshoot and oscillations is due to the nonlinearity of the system. A large overshoot may result in the inadequacy of the linearized model and instability of the real system.

Essentially, most controller tuning or designing processes are multi-objective problems. In our case, the first objective function is the overshoot PO and the second one is the settling time $T_{s}$ while the PID parameters $\left(K_{p},K_{i},K_{d}\right)$ are selected as problem decision variables. The properties of magnetic levitation depend on parameters of the controller. In industry settings, controller parameters obtained using multi-objective genetic algorithms lead to a reduction of energy consumption, improved performance overall or and increased production rate [51,52].

In order to compare the effectiveness of the developed algorithm, we used common minimum search methods. One of them is the NSGA-II (Non-dominated Sorting Genetic Algorithm) with overshoot PO,% and settling time $T_{s}$ as the two objective functions. NSGA-II deals with a set of solutions simultaneously which improves the computational speed. Quite often this feature allows it to select several solutions of Pareto set in a single run of the algorithm [53].

To investigate this further, a search using the PSO (Particle Swarm Optimization) algorithm was performed with overshoot PO,% being the single objective function. PSO is an optimization method, which iteratively tries to improve a solution with regard to a given measure of quality. A particle is usually an element in some vector space—in our case $\left(K_{p},K_{i},K_{d}\right)$. PSO performs searching via a swarm of particles that updates each iteration. Using simple formulae, each particle moves in a direction depending on its previous best position and the best position among all of the particles in the whole swarm. Optimization ends if the relative change in the objective value over the last iterations is less than a defined function tolerance [54].

**Figure 13:**
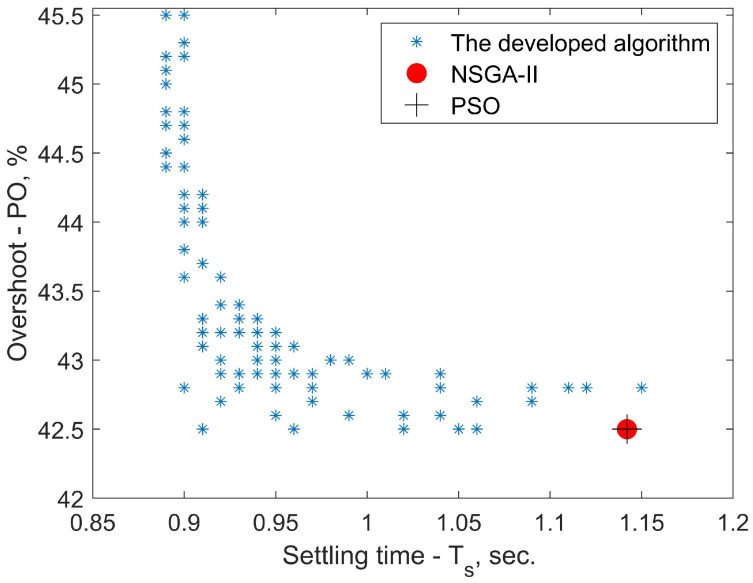
A comparison between the developed algorithm and genetic search methods. With the used boundaries for the decision variables $\left(K_{p},K_{i},K_{d}\right)$ the developed algorithm proved to be insensible to the local minima trapping effect as it showed multiple solutions on the Pareto set. Combined with fast computations, this makes for an optimal choice for a design method involving transient response analysis.

For the NSGA-II and PSO the Global Optimisation Toolbox in Matlab has been used. The decision variables of the search (the controller parameters) were limited to $K_{p}\in \left[0,200\right],\;K_{p}\in \left[0,250\right],\;K_{p}\in \left[0,10\right]$ due to hardware limitations (23). Figure 13 shows that NSGA-II outputs one point on the Pareto-front. This result stays the same after around one thousand total function evaluations. Usually in these situations, a rule of thumb is to find the single-objective minima to start the algorithm search from. By doing so, one may be able to obtain a wider Pareto set by avoiding a potential local minima. It is possible, however, that one still ends up with a single point, which means there is only one feasible Pareto point. In this study, starting from various points in the controller parameter space did not yield a different result.

The search using PSO yielded the same solution as seen in Figure 13. With this, we may conclude that there is no tradeoff curve (Pareto front) because all the objectives are minimized at the same point. The developed algorithm, on the other hand, was not affected by any local minima. While it is not a genetic-based algorithm the computational time was around the same as for the other two methods (simulations showed that all of the three algorithms required around $10^{3}$ function evaluations).

### 4.2. Experimental Verification

To provide an experimental confirmation, as well as to prove the effectiveness of the designed algorithm, we compared its results to the measurements using the magnetic levitation system, thoroughly described in the corresponding section. First, we made the magnet levitate steadily at $z_{0}=-25$ mm with an empirical tune of $\left(K_{p},K_{i},K_{d}\right)=\left(150,45,6.25\right)$. There are two main reasons behind this. First is to standardize the initial conditions before the start of each experiment. Second is for the system to be more comparable with the linearization. Then, we inserted new PID parameters in the microcontroller software to make sure the system is still stable. If it was, we forced the permanent magnet to rise Δz=+1 mm, while recording the transient response.

**Figure 14:**
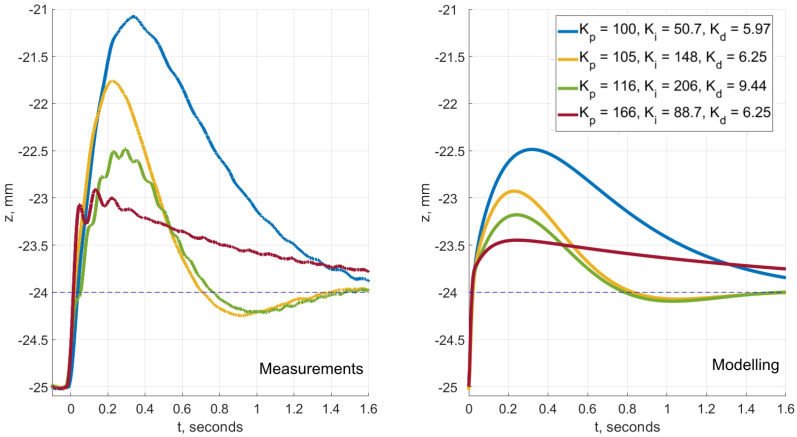
A comparison of a sequence of measured and modeled responses with reducing overshoot. Overall, the modeling is consistent with the experiments. The key feature influencing the compliance of the data is the accuracy of the used expression for the magnetic force. As expected, with larger values of overshoot (blue and yellow curves) the model becomes less accurate. The difficulties involving the nonlinearity identification of the magnetic force of the coil applied to the permanent magnet in a form of a small cylinder are discussed in the corresponding section.

The comparison between the experiments and the simulations performed by the algorithm can be seen in Figure 14. The transients are between −25 mm and −24 mm. Both states are very close to the point of linearization, so the system performs fairly well and the results agree with the modeling. The relative values of overshoots related to the measured curves correspond with those obtained by the algorithm. The step responses with larger overshoot are expected to diverge more from the modeled ones. The divergence increases as the permanent magnet travels further from the equilibrium point at which the magnetic force function was expanded into the Taylor series.

For this research, as was shown before, Formula (5) has an approximation with the sum of squared errors lower by several orders of magnitude than the most commonly used formulas. However, the linearization still adds some inaccuracy. The real magnetic force changes with distance *z* by a different law. It is quite difficult to carefully estimate a finite size coil’s magnetic force applied to a permanent magnet, when the dimensions of the coil are comparable to the distance to the permanent magnet. Another obstacle would be taking into account the shape of the permanent magnet. These are serious mathematical problems that go beyond this research’s goal which is to demonstrate the capabilities of the designed controller tuning and system design algorithm. For the purpose of this research, it is sufficient to use the approximation we presented in Section The Magnetic Levitation System—Figure 14 reflects that. We also discuss the possibility of improving the accuracy of the modeling in the following section.

### 4.3. The Multi-Objective Problem for Optimal Coil Parameters

Here we show how, by taking into account more information about the magnetic levitation system and varying one or more of the coil’s parameters, one may find their optimal values. It is possible to use the data provided by the algorithm as a criterion for optimization. One may look at it as a Monte-Carlo method of sorts. Here we discuss an example of this method’s realization. Before, as an approximation we used formula (5) for the magnetic field of the coil:

$$B_{z}=\frac{\mu_{0}}{2\pi }i\frac{\pi R^{2}}{{\left(z^{2}+R^{2}\right)}^{3/2}}.$$

This is, however, the magnetic field caused by just one of the rings within the coil. For that reason, let us perform an integration over the length of the coil. We should point out, though, that any mistake in the formulas at this stage will result in a wrong analysis overall. So extra caution should be taken to keep the expressions correct. In our setup, the permanent magnet is situated below the coil, which means z<0. That is why

$$B_{z\;coil}=\frac{\mu_{0}}{2\pi }ni\int_{0}^{L}\frac{\pi R^{2}\;dl}{{\left({\left(l-z\right)}^{2}+R^{2}\right)}^{3/2}}=$$

(24) $$=\frac{\mu_{0}}{2}ni\left(\frac{L-z}{{\left(L-z\right)}^{2}+R^{2}}+\frac{z}{z^{2}+R^{2}}\right),$$

where *L* is the length of the coil and *n* is the density of winding turns per unit length. To get the explicit expression of the magnetic force in (4), the final step is to perform the differentiation of this expression.

(25) $${\frac{\partial B_{z}}{\partial z}}_{coil}=\frac{\mu_{0}}{2}ni\left(\frac{{\left(L-z\right)}^{2}-R^{2}}{{\left({\left(L-z\right)}^{2}+R^{2}\right)}^{2}}+\frac{R^{2}-z^{2}}{{\left(z^{2}+R^{2}\right)}^{2}}\right),$$

The linearization of the process involved requires a Taylor transformation, after which Newton’s equation is converted to

(26) $$m\frac{d^{2}\hat{z}}{dt^{2}}=\frac{\partial F_{z}}{\partial z}\left(i_{0},z_{0}\right)\cdot \hat{z}+\frac{\partial F_{z}}{\partial i}\left(i_{0},z_{0}\right)\cdot \hat{i},$$

where

$$\frac{\partial F_{z}}{\partial z}\left(i_{0},z_{0}\right)=-2\mu_{z}\frac{\mu_{0}}{2}ni_{0}\;\times$$

$$\times \left(\frac{\left(L-z_{0}\right)\left(3R^{2}-{\left(L-z_{0}\right)}^{2}\right)}{{\left({\left(L-z_{0}\right)}^{2}+R^{2}\right)}^{3}}+\frac{z_{0}\left(3R^{2}-z_{0}^{2}\right)}{{\left(z_{0}^{2}+R^{2}\right)}^{3}}\right),$$

$$\frac{\partial F_{z}}{\partial i}\left(i_{0},z_{0}\right)=\mu_{z}\frac{\mu_{0}}{2}n\;\times$$

(27) $$\left(\frac{{\left(L-z_{0}\right)}^{2}-R^{2}}{{\left({\left(L-z_{0}\right)}^{2}+R^{2}\right)}^{2}}+\frac{R^{2}-z_{0}^{2}}{{\left(z_{0}^{2}+R^{2}\right)}^{2}}\right).$$

Now the acquired transfer function has the length of the coil *L*, the radius of the coil *R* and the density of winding turns per unit length *n* within the transfer function. This opens a whole new mathematical problem of optimizing these parameters for best performance. For this, our algorithm with its rapid calculations is a great tool for optimization. This simulation data can be the basis for a multi-objective optimization to determine the best values for these parameters. First, we postulate an optimization criterion which may consist of one feature of the step response or several of them. Then, the algorithm provides characteristics of thousands of modeled step responses that can be visually represented as in Figure 13 using radar charts or a Pareto-optimal front.

## 5. Conclusions

In this study, we presented a framework for designing control systems. For this purpose, an algorithm was created which determines the key features of simulated transient responses. With modern computing power, this algorithm is suitable for multi-objective optimization tasks such as nominal parameters of complicated mechanical systems. The expected curve form of regular stable transient responses opens a possibility of fast curve analysis using the developed algorithm. This, in turn, opens the possibility of collecting large simulation data samples, which can be applied to solve different multi-optimization problems such as optimal coil parameters. A comparison with common genetic search methods showed the competitiveness of the designed algorithm.

With the crafted magnetic levitation system as a means of testing, we were able to show the algorithm’s versatility. Specific features were discussed, such as linearizing the magnetic levitation force on the permanent magnet as well as curve fitting of the measured data. In this case, the main focus was reducing the overshoot, for which the proposed method was able to provide numerous suitable sets of controller parameters. The transient response behavior, as well as values of overshoot related to the measured curves, corresponded well with those obtained by the simulation. Therefore, this method proved to be highly effective for pre-production purposes.

The main benefits of the developed algorithm can be summarized. Coding, applicable for any programming language. An important feature is its immunity to the effect of local minima trapping. As shown in Figure 13, the developed algorithm in the given conditions proved to be versatile and provided multiple suitable solutions to the multi-objective problem between objective functions of overshoot and settling time. In addition, we showed how the algorithm automatically adjusts the size of the time step of the simulated signal to meet the conditions of the Nyquist–Kotelnikov–Shannon theorem. As we mentioned, this feature is particularly important for open-loop unstable systems (such as the magnetic levitation system) since unsupervised large oscillations may result in unwanted sideways irregularities in motion and instability of the whole controlled system. This method is a basis for solving a multi-objective problem of optimal coil dimensions and proportions. Additional accuracy in the nonlinearity identification process will lead to a wider optimization problem of not only the controller parameters but also key features of any magnetic levitation system.

## Figures and Tables

**Table 1:** Magnetic force $F_{m}$ in millinewtons [mN] in relationship to the current *i* in Amperes [A] and the permanent magnet position *z* in millimeters [mm].

*i* A	0 A	0.2 A	0.4 A	0.6 A	0.8 A	1.0 A
z=−15 mm	0 mN	52.0 mN	101.0 mN	151.1 mN	200.1 mN	250.2 mN
z=−20 mm	0 mN	32.4 mN	63.8 mN	94.2 mN	126.5 mN	156.0 mN
z=−25 mm	0 mN	23.5 mN	45.1 mN	65.7 mN	86.3 mN	106.9 mN
z=−30 mm	0 mN	14.7 mN	28.4 mN	42.2 mN	54.9 mN	68.7 mN
z=−35 mm	0 mN	9.8 mN	18.6 mN	28.4 mN	38.3 mN	48.1 mN
z=−40 mm	0 mN	6.9 mN	13.7 mN	20.6 mN	27.5 mN	33.4 mN

**Table 2:** The results of data analysis using sum of squared errors.

Function	$-\mathrm{aiz}/{\left(z^{2}+R^{2}\right)}^{5/2}$	$\mathrm{ai}/z^{2}$	ai/z	$\mathrm{ai}/z^{3}$
SSE	$5.6\times 10^{-5}$	0.0026	0.017	0.022

**Table 3:** Sensor circuit output $U_{s}\left[V\right]$ in relationship to the permanent magnet position z[mm].

*z* [mm]	$U_{s}$ [V]
−15	3.32
−20	1.20
−25	0.504
−30	0.219
−35	0.135
−40	0.072

**Table 4:** Step-response characteristics provided by the tuning algorithm. The value of $OA_{max}$ provides us with the scale of oscillations. If the number of local extremums k≤1, then this value is obviously not defined.

$K_{p}$	$K_{i}$	$K_{d}$	$T_{s},$ (s)	*k*	${\mathrm{OA}}_{\max}$	PO	$t_{\min},$ (s)
⋯	⋯	⋯	⋯	⋯	⋯	⋯	⋯
223	875	9.58	0.49	1	—	61.3	0.0026
534	938	6.75	0.74	3	0.59	80.7	0.0032
⋯	⋯	⋯	⋯	⋯	⋯	⋯	⋯
85.1	171	3.67	1.41	4	2.7	129	0.0047
224	391	8.15	0.26	1	—	63.5	0.0030
⋯	⋯	⋯	⋯	⋯	⋯	⋯	⋯

## Data Availability

The data used in this research is available from the corresponding author upon reasonable request.
